# Gossypol Promotes Degeneration of Ovarian Follicles in Rats

**DOI:** 10.1155/2014/986184

**Published:** 2014-07-23

**Authors:** Ivana Cristina Nunes Gadelha, Michelly Fernandes de Macedo, Sílvia Catarina Salgado Oloris, Marilia Martins Melo, Benito Soto-Blanco

**Affiliations:** ^1^Programa de Pós-graduação em Ciência Animal, Universidade Federal Rural do Semiárido, BR 110 Km 47, 59628-360 Mossoró, RN, Brazil; ^2^Fundação Ezequiel Dias (FUNED), Rua Conde Pereira Carneiro 80, 30510-010 Belo Horizonte, MG, Brazil; ^3^Departamento de Clínica e Cirurgia Veterinárias, Escola de Veterinária, Universidade Federal de Minas Gerais, Av. Antônio Carlos 6627, 30123-970 Belo Horizonte, MG, Brazil

## Abstract

The present study aimed to determine if gossypol interferes with ovarian follicles in rats. Twenty-four female Wistar rats were assigned to two equal groups: one control group and the other dosed with gossypol (25 mg/kg/day, subcutaneously) for 15 days. Ovarian follicles were histologically classified according to the stage of development and as normal or atretic. Gossypol treatment reduced the length of estrous with an increase in the duration of the diestrus phase. This compound was responsible for reduced serum levels of T4 and progesterone. Treatment with gossypol was responsible for a significant reduction in the number of normal ovarian follicles and a significant increase in the number of atretic follicles, both in all stages of development. Thus, treatment of rats with gossypol was responsible for reduction in the number of viable follicles and changes in hormone levels that resulted in interference of the estrous cycle.

## 1. Introduction

Gossypol (2,2-bi(8-formyl-1,6,7-trihydroxy-5-isopropyl-3-methylnaphthalene)) is a polyphenolic compound produced by pigment glands in roots, stems, leaves, seeds, and flower buds of cotton (*Gossypium* spp). The function of this compound is to deter various insects from feeding on the plant [1, 2]. Signs of gossypol toxicosis include decreased growth rate, anorexia, labored breathing, and dyspnea [2]. Other effects include damage to hepatocytes [3, 4] and lymphocyte cytotoxicity leading to immunodeficiency [5–7].

The male reproductive toxicity of gossypol has been reported in many studies [8]. Gossypol inhibits spermatogenesis by decreasing the sperm count and spermatozoid motility and viability. The male infertility effect is caused by impaired sperm motility, decreased sperm concentrations, and specific mitochondrial injury to the sperm tail and damage to the germinal epithelium [9–12]. The gossypol-mediated spermatozoid disturbance mechanism includes inhibition of the release and utilization of ATP by sperm cells [13]. Furthermore, gossypol inhibits calcium influx and Mg-ATPase and Ca-Mg-ATPase activity in spermatozoid plasmatic membranes [14]. Abnormal spermatozoids are produced because gossypol produces ultrastructural alterations in the nuclear membrane, endoplasmic reticulum, and mitochondria [15, 16]. In cultured Sertoli cells from pigs, gossypol also decreased cellular oxidase activity and DNA damage [17]. Reduced nuclear expression of androgen receptors was observed in Leydig cells, Sertoli cells, and myoid cells from rats fed gossypol-rich cottonseed flour [18]. However, such effects are reversible after the cessation of gossypol exposure [19].

Even though gossypol has well-known toxic effects in the male reproductive system, there are few studies on its effects on female reproduction. Female exposure to gossypol has been associated with interference of the estrous cycle, pregnancy, and early embryonic development [1, 2]. Gossypol has been shown to affect rodent estrous cycles [20, 21] and pig granulosa cell function [22]. Gossypol affected ovarian steroidogenesis* in vitro* [23, 24] as well as bovine oocyte cumulus expansion and nuclear maturation [24]. The present study aimed to determine whether gossypol interferes with the ovarian folliculogenesis in rats. Our work also proposes a model for determining the effects of toxic substances on ovarian metabolism by ovarian follicle classification and quantification.

## 2. Material and Methods

### 2.1. Animals

Female Wistar rats, 60 to 70 days old, weighing approximately 120 g each, from the Animal Sciences Department, UFERSA, Mossoró, RN, Brazil, were used. During the entire study period, the rats were housed in plastic cages under controlled environmental conditions with a 12 h light/dark cycle and at a temperature of 24 ± 3°C. Regular rodent chow (Labina, Purina, São Lourenço da Mata, PE, Brazil) and tap water were provided* ad libitum*. Animal studies have been approved by the Institutional Animal Care and Use Committee at the Universidade Federal Rural do Semi-Árido/UFERSA (process number 23091.000690/2012-72) and have been performed in accordance with the ethical standards laid down in the 1964 Declaration of Helsinki and its later amendments.

### 2.2. Experimental Design

The estrous cycle of the rats was monitored twice a day by vaginal smear for seven consecutive days; only rats that exhibited regular estrous cycles were used in the experiment. Twenty-four female rats were randomly and equally distributed into two groups: a control group (injected with saline solution subcutaneously) and a group dosed with gossypol (25 mg/kg/day subcutaneously) for 15 consecutive days. Rats were dosed with (+/−) gossypol acetic acid (Fluka, G4382) with purity about 95%.

Vaginal smears were carried out on each animal every day between 7:00 and 9:00 a.m. and again between 5:00 and 6:00 p.m. The smears were collected by lavage with 20 *μ*L of 0.9% NaCl solution, spotted thinly on a microscope slide, and evaluated under a light microscope. The cell counts and morphology were used for the determination of the estrous cycle phases, characterized as proestrus, estrus, metestrus, and diestrus [25].

On the day after the last dosing, all rats were deeply anesthetized by intraperitoneal injection of xylazine (5 mg/kg) and ketamine (60 mg/kg) to collect the ovaries, uterus, thyroid glands, and blood samples from the vena cava. Formalin-fixed, paraffin-embedded tissue samples were sliced into 5 *μ*m thick sections and stained with hematoxylin and eosin (H&E) for histological analysis.

### 2.3. Hormonal Analysis

Frozen sera were used for the determination of the serum levels of estradiol, progesterone, luteinizing hormone (LH), thyroxine (T4), triiodothyronine (T3), and thyroid stimulating hormone (TSH). Hormone levels were analyzed by an ELISA automated analyzer (Elisys Uno, Human, Wiesbaden, Germany) with specific kits (RPC Diagnostic Systems, Nizhny Novgorod, Russia).

### 2.4. Morphological Analysis of the Ovarian Follicles

**Figure 1:**
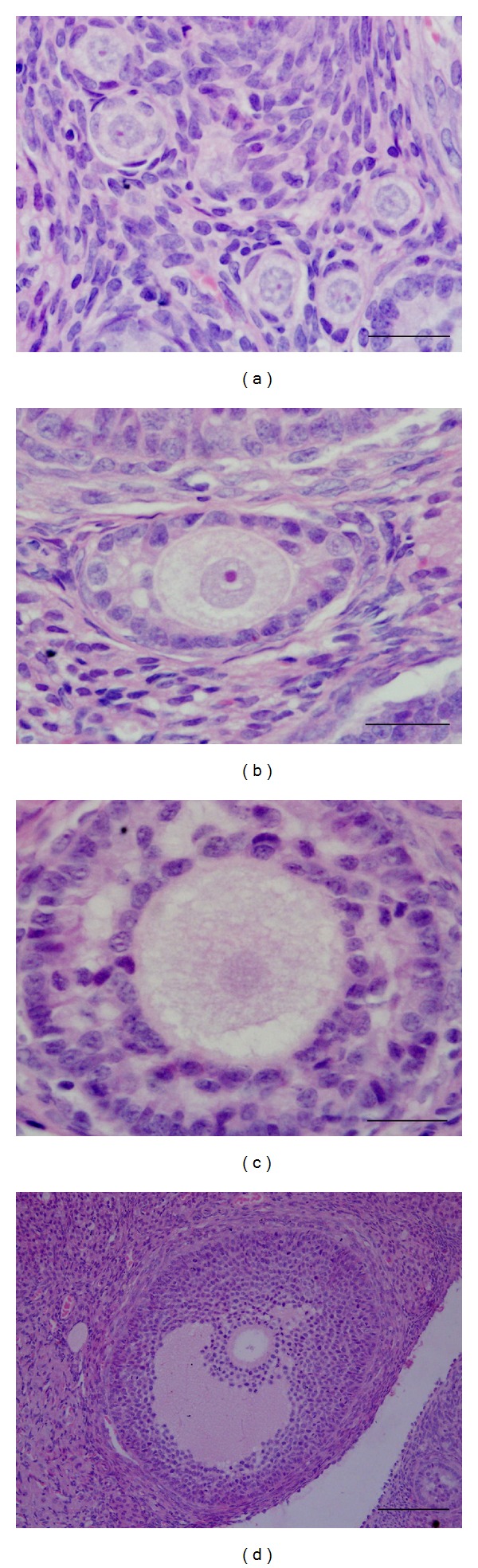
Ovaries from control rats showing normal (a) primordial, (b) primary, (c) secondary (H&E, bar = 25 *μ*m), and (d) antral follicles (H&E, bar = 100 *μ*m).

The ovaries from all rats were collected and fixed in 10% buffered formalin. Five-micron thick paraffin-embedded sections were collected at 60 *μ*m intervals throughout the tissue and stained with H&E. Histological analysis, including qualitative and quantitative assessment of ovarian follicles, was performed. Follicles were classified according to the stage of development as primordial, primary, secondary, or antral [26]. Primordial follicles were defined as those with one layer of flattened granulosa cells, primary follicles presented one layer of cuboidal granulosa cells, secondary follicles presented two layers of cuboidal granulosa cells, and antral follicles presented the antral cavity (Figure 1).

Follicles were also classified as normal or atretic. Normal follicles presented a regular shape and well-organized granulosa cells, without signs of atresia. Atretic follicles were characterized by retracted oocytes, a pyknotic nucleus, discontinued basement membrane, and disorganized granulosa cells [27, 28].

### 2.5. Statistical Analysis

The obtained data were statistically analyzed using R software (version 3.0.0). Data normality was evaluated using a Shapiro-Wilk test and the homogeneity of variance was evaluated using an *F* test. Welch's *t*-test was employed and *P* values < 0.05 were considered statistically significant.

## 3. Results

No clinical signs of poisoning were observed in any of the rats. There was no significant difference in body weight between the groups on the first and last days of the experiment, but body weight gain was significantly (*P* < 0.05) reduced in rats dosed with gossypol (Table 1). Furthermore, the treatment with gossypol significantly (*P* < 0.05) increased the length of the diestrus stage (Table 1).

At necropsy, hydrometra was observed in five of the rats dosed with gossypol and in one rat from the control group. No histological lesions were found in the thyroid glands or the uterus from any of the rats.

Our results also verified that gossypol was responsible for hormonal interference, characterized by significantly (*P* < 0.05) reduced serum levels of T4 and progesterone and a significantly (*P* < 0.05) increased T3/T4 ratio (Table 2). Conversely, gossypol treatment did not affect the serum levels of T3, TSH, LH, and estradiol.

**Figure 2:**
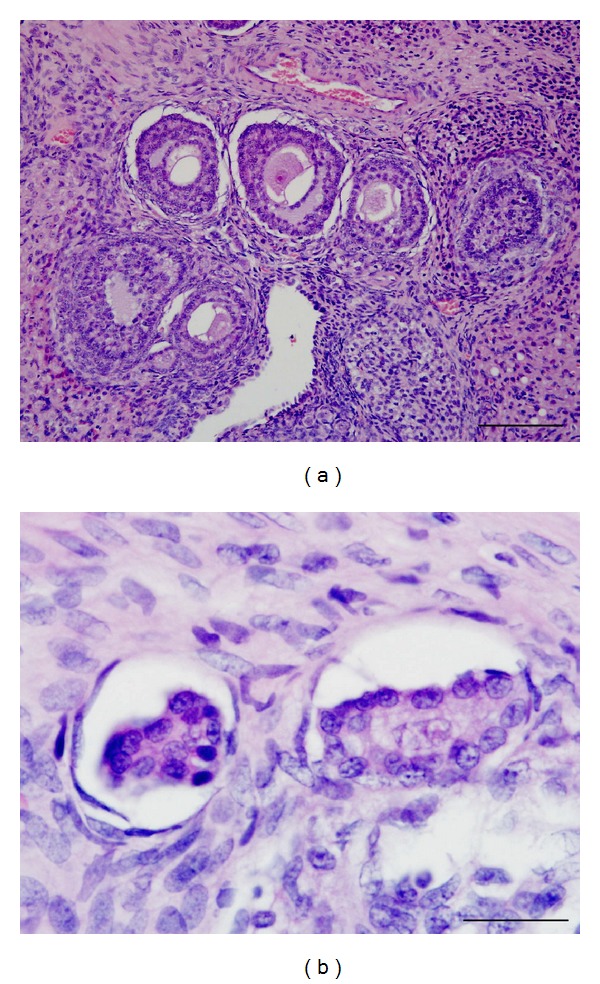
(a) Ovaries from gossypol treated rats showing atretic primordial, primary, and secondary follicles (H&E, bar = 100 *μ*m). (b) Atretic primordial and primary follicles from a gossypol-dosed rat (H&E, bar = 25 *μ*m).

Additionally, treatment with gossypol was responsible for a significant (*P* < 0.05) reduction in the number of normal ovarian follicles and a significant (*P* < 0.05) increase in the number of atretic follicles, both in all stages of development (Table 3 and Figure 2). On average, the ovaries of female rats in the control group had 83.3% normal follicles and 16.7% atretic follicles, whereas rats treated with gossypol had normal and atretic follicle incidence rates of 36.6% and 63.4%, respectively.

## 4. Discussion

We found that gossypol treatment resulted in increase in the length of the diestrus and metestrus stages (about 116% and 350% of controls, resp.) and reduction in the length of the proestrus and estrus stages (both about 81% of controls). Interferences on estrous cycle were observed by others [20, 21]. The administration of 20 mg/kg of gossypol acetic acid orally for 60 days [20] and of 25 mg/kg gossypol acetic acid via intramuscular injection for 3 to 5 days [21] caused prolonged and irregular estrous cycles in rats. Female rats dosed with 60 mg/kg gossypol acetic acid for 30 days showed a reduced number of estrus cycles, which did not occur when they were dosed with 40 mg/kg [29]. Similarly to our study, the percentage of animals in diestrus was increased in rats dosed with 5 or 10 mg gossypol/kg/day subcutaneously for 20 days [30]. Conversely, gossypol treatment has been shown to have no effect on ovulation in rats [31] and did not alter estrous cycles in hamsters [32].

Our results verified that gossypol is responsible for hormonal interference, as evidenced by reduced serum levels of progesterone and T4 and an increased T3/T4 ratio. Reduced serum levels of progesterone were reported in female rats that received 25 mg/kg gossypol acetic acid via intramuscular injection for 3 to 5 days [21] and in pregnant rats dosed orally with 60, 90, and 120 mg/kg/day gossypol acetic acid for the first 10 days after coitus [33]. Data from the* in vitro* studies indicate that gossypol inhibited the basal and stimulated progesterone secretion levels in luteal cells [23, 34], granulosa cells from small follicles [35], and oocyte-cumulus complexes isolated from large antral porcine follicles [36]. Several mechanisms may be involved in the inhibition of progesterone synthesis, including the inhibition of adenylate cyclase [23, 34] and 3*β*-hydroxysteroid dehydrogenase [37], as well as by nitric oxide generation [35]. Thus, the interference of ovarian steroidogenesis may be the mechanism by which gossypol affects the estrous cycle.

Gossypol is also an antithyroidal compound [38, 39]. Rats from the present study showed reduced serum levels of free T4 and had an increased T3/T4 ratio. Earlier studies with male [38] and female [39] rats showed decreased blood levels of free T4 and T3 levels after dosing with gossypol acetic acid. Moreover, the histopathological evaluation of thyroid glands from male rats revealed follicular degeneration and atrophy [38], but no histological alteration was observed in thyroid glands in our study. Reduced T3 levels were also observed in rabbits, but this reduction was reverted when dosing was discontinued [10]. However, gossypol dosing resulted in increased T3 serum levels without affecting T4 in rats [40] and sheep [41]. The thyrotropic cells in the pituitary gland, which specializes in TSH synthesis and secretion, showed hypertrophy, hyperplasia, and degranulation after gossypol acetate dosing in rats [42]. This finding differs from our results, which indicated that TSH levels were not affected by gossypol treatment. Although these findings are conflicting, there is a general consensus that gossypol affects thyroid gland metabolism, but the mechanism is uncertain.

The treatment with gossypol was also responsible for both a significant reduction in the number of normal follicles and a significant increase in the number of atretic follicles in all stages of development. On average, the ovaries of the rats in the control group had 83.3% normal follicles and 16.7% atretic follicles, whereas the rats treated with gossypol had 36.6% normal follicles and 63.4% atretic follicles. All types of normal follicle cell types are reduced about a third and the atretic follicle cell types are increased about three times. The ovarian weight was decreased in rats dosed with 5 or 10 mg gossypol/kg/day subcutaneously for 20 days [30]. Conversely, no pathological lesions were found in the ovaries of female rats dosed with 40 or 60 mg/kg gossypol acetic acid for 30 days [29]. However, in both studies [29, 30] the ovarian follicles were not classified not counted. Thus, the classification and counting of ovarian follicles used in our study may be a useful tool for studying compounds that potentially affect female gametogenesis.

In female mammals, the number of ovarian primordial follicles is fixed at the time of birth. The primordial follicles grow into primary, secondary, and antral follicles in a continuous and nonreversible process. The damage to primary, secondary, and antral follicles may lead to temporary infertility when the primordial follicles are not affected. On the other hand, the injury to primordial follicles may result in permanent infertility because of the eventual depletion of the pool of these follicles [43]. Therefore, it is feasible to speculate that gossypol exposure may reduce the ovarian reserve of follicles, impairing female fertility. However, gossypol seems to not be a potential drug to promote female castration because of its toxicity, which in our study was evidenced by body weight loss.

It was observed that gossypol inhibited steroidogenic activity and redox status and stimulated the vascular endothelial growth factor production in an* in vitro* experiment with porcine granulosa cells [22]. Furthermore, gossypol showed cytotoxic and apoptotic activity in ovarian carcinoma cell lines [44, 45]. Due to increased follicular atresia, the folliculogenesis could be severely degraded by the action of this compound, dramatically reducing the conception rate of exposed females. In fact, female rats that received 20 mg/kg/day of gossypol acetic acid for 60 days presented delayed mating and a reduced rate of pregnancy and number of viable embryos when compared to controls [20]. Additionally, it is likely that exposed females will experience early reproductive senescence.

An additional interesting observation made at necropsy was the occurrence of hydrometra in five rats from the gossypol treated group and one rat from the control group. High incidence of infertility and atrophy of the uterus was observed in women exposed to gossypol through the ingestion of cottonseed oil in China [8]. Thus, the increased frequency of hydrometra must be one of the effects of gossypol, which likely contributes to the infertility of females exposed through the ingestion of cotton seed.

In conclusion, treatment with gossypol was responsible for interference of the estrous cycle in the rats. This interference involved a reduction in the number of viable follicles as well as hormonal changes, which might negatively impact female reproduction. The overall results from the present study show the relevance of ovarian follicle classification and quantification in the determination of estrous cycling. This is pertinent for future studies that aim to determine the effects of toxic substances on ovarian metabolism.

## Figures and Tables

**Table 1:** Body weight, body weight gain (in grams), and length (in hours) of the estrous cycle phases of female rats dosed with gossypol (25 mg/kg/day) or 0.9% saline (control group) subcutaneously for 15 consecutive days.

	Control (*n* = 12)	Gossypol (*n* = 12)	*P* value^1^
Body weight (g)			
Day 0	190.4 ± 4.32	192.2 ± 7.62	n.s.
Day 15	197.5 ± 2.91	186.7 ± 6.15	n.s.
Body weight gain (g)	7.08 ± 2.07	−5.58 ± 2.43	0.0006792
Diestrus (h)	44.0 ± 1.71	51.0 ± 2.15	0.01875
Metestrus (h)	1.00 ± 0.67	3.50 ± 1.73	n.s.
Estrus (h)	24.5 ± 1.88	20.0 ± 1.71	n.s.
Proestrus (h)	26.5 ± 1.16	21.5 ± 2.39	n.s.

^1^Welch's *t* test.

n.s.: not significant (*P* > 0.05).

**Table 2:** Serum concentrations of estradiol, progesterone, luteinizing hormone (LH), thyroxine (T4), triiodothyronine (T3), and thyroid stimulating hormone (TSH) in female rats dosed with gossypol (25 mg/kg/day) or 0.9% saline (control group) subcutaneously for 15 consecutive days.

	Control (*n* = 12)	Gossypol (*n* = 12)	*P* value^1^
T4 (ng/dL)	2.21 ± 0.10	1.80 ± 0.12	0.01446
T3 (pg/mL)	5.68 ± 0.41	7.73 ± 1.07	n.s.
T3/T4 ratio	2.62 ± 0.19	4.62 ± 0.77	0.02635
TSH (*µ*IU/mL)	4.00 ± 1.76	3.91 ± 1.75	n.s.
Progesterone (ng/mL)	48.3 ± 7.61	24.5 ± 4.16	0.01317
LH (mIU/mL)	0.22 ± 0.04	0.21 ± 0.04	n.s.
Estradiol (pg/mL)	10.7 ± 3.83	16.7 ± 4.53	n.s.

^1^Welch's *t* test.

n.s.: not significant (*P* > 0.05).

**Table 3:** Ovarian follicle populations in female rats dosed with gossypol (25 mg/kg/day) or 0.9% saline (control group) subcutaneously for 15 consecutive days.

Follicles	Control (*n* = 12)	Gossypol (*n* = 12)	*P* value^1^
Number of normal follicles			
Primordial	341.8 ± 25.6	122.6 ± 20.1	<0.0001
Primary	115.5 ± 14.8	49.0 ± 5.03	0.0008635
Secondary	62.6 ± 11.3	20.1 ± 4.54	0.003426
Antral	55.8 ± 6.52	14.5 ± 3.71	<0.0001
Total	575.6 ± 51.8	206.2 ± 27.9	**<0.0001**

Number of atretic follicles			
Primordial	66.3 ± 10.7	168.3 ± 24.1	0.001489
Primary	22.5 ± 2.32	65.8 ± 6.99	<0.0001
Secondary	22.2 ± 4.26	71.7 ± 9.44	0.00023
Antral	8.08 ± 1.52	39.6 ± 3.95	<0.0001
Total	119.1 ± 12.8	345.4 ± 32.8	**<0.0001**

Proportion of follicles (in %)			
Normal	82.1 ± 2.05	36.6 ± 1.73	<0.0001
Atretic	17.8 ± 2.05	63.4 ± 1.73	<0.0001

Number of total follicles			
Total	694.7 ± 54.5	551.6 ± 57.2	n.s.

^1^Welch's *t* test.

n.s.: not significant (*P* > 0.05).
